# Economic gradient of onset of disability in India

**DOI:** 10.1186/s12889-021-10826-5

**Published:** 2021-04-21

**Authors:** Radhe Shyam Mishra, Sanjay K. Mohanty, Jack Cordes, Umakanta Sahoo, Rajeev R. Singh, S. V. Subramanian

**Affiliations:** 1 International Institute for Population Sciences, Govandi station road Deonar, Mumbai, 400088 India; 2 Department of Fertility Studies, International Institute for Population Sciences, Mumbai, India; 3 Department of Epidemiology, Harvard T.H. Chan School of Public Health, Boston, MA USA; 4 Harvard Centre for Population and Development Studies, Harvard University, Cambridge, MA USA

**Keywords:** Disability, Economic gradient, Onset, type of disability, India

## Abstract

**Background:**

Disability in India is associated with increasing non-communicable diseases, rising longevity, and increasing accidents and injuries. Though studies have examined prevalence, patterns, and socioeconomic correlates of disability, no attempt has been made in estimating age of onset of disability in India.

**Objective:**

This paper investigates the economic gradient of age of onset of locomotor, visual, hearing, speech, mental retardation, mental illness, and other disabilities in India.

**Method:**

We use nationally representative data of 106,894 disabled individuals from the 76th round of National Sample Survey (NSS), 2018. Descriptive statistics, kernel density, Kaplan-Meier survival curves, and linear regression models are used in the analysis.

**Result:**

The disability rate in India was 2184 per 100,000 persons. The disability rate was highest for locomotor (1353) followed by hearing (296), visual (234), speech (228), mental retardation (158), and mental illness (131). Over 85% of mental retardation and 80% of speech disabilities occur at birth, while 82% of locomotor and 81% of visual disabilities occur after birth. Among those who had disability after birth, the median age for mental retardation was 2 years followed by mental illness (28 years), speech (29 years), locomotor (42 years), visual (55 years), and 56 years for hearing disability. Adjusting for socioeconomic covariates, the age of onset of locomotor and speech disabilities among the poorest individuals were 7 and 11 years earlier than the richest, respectively.

**Conclusion:**

The economic gradient of onset of locomotive and speech disabilities are strong. The age of onset of disability was earliest for mental retardation followed by mental illness and speech disability.

**Supplementary Information:**

The online version contains supplementary material available at 10.1186/s12889-021-10826-5.

## Introduction

Disability is an emerging public health, economic, and social challenge worldwide. Globally, over 150 million people have any form of disability and 6 million of them have severe disability [1]. Disability varies largely within and between countries, regions, and by socioeconomic and demographic characteristics owing to disease burden, stages of demographic transition, and definitional differences [2–4]. Efforts for the rights of disabled persons has intensified in recent decades. The convention on the rights of persons with disabilities [5] was featured in the sustainable development goals agenda, which aims to ensure a healthy life and to promote well-being for all at all ages [6–8].

With more countries moving towards later stages of the demographic transition, life expectancy has been increasing across all age groups. Though people are living longer, many are living with disease and deformity. Along with the demographic transition, late stages of the epidemiological transition are characterised by increases in non-communicable diseases (NCDs), which are the leading causes of mortality and morbidity worldwide [9] and are associated with higher risk of disability [10]. The onset of NCDs is increasingly at younger ages and making people vulnerable to disability [11] earlier in life. Furthermore, poor socioeconomic conditions and unmet health care needs make duration of disability longer [10]. Compared to persons without disabilities, persons with disabilities are more prone to adverse socioeconomic outcomes, poor health, lower educational attainment, higher unemployment and underemployment, increased poverty, increased physiological stress, and decreased accessibility to services [12, 13]. Disability may increase the risk of poverty through lack of employment and education opportunities, lower wages, and higher cost of living [3, 14].

The onset of disability, defined as the age of occurrence of disability, is associated with a set of biological, social, economic, and environmental factors. Studies have shown that disability is more likely to occur at birth and early childhood as well as in old age [12, 15, 16]. For example, in India, one-third of disabilities occur at birth [17]. Disability at birth may be caused by many factors: genetics, care during pregnancy, complications during birth, and infections at time of birth among other factors [18, 19]. Disability following birth is largely due to varying socioeconomic, environmental, and demographic factors [20, 21]. The onset of disability is changing and disability has been increasing across all ages, although disability onset is clustered at younger and older ages [22]. Increasing disability is associated with increasing size and proportion of the elderly population, chronic health diseases, and accidents and injuries [23–25].

India with 1.21 billion population (the second most populous country) is estimated to have 23 million disabled persons in 2011 [26]. About 2.2% of the country’s population were disabled and locomotor disability accounts one-fifth of disabled (20.3%) followed by hearing (19.0%), seeing (18.5%), other disability (18.0%), multiple disability (8.0%), speech (7.5%), mental retardation (5.5%), and mental illness (2.7%). Disability is higher among females than males and higher in older ages. India is also experiencing a rapid increase in its elderly population; about 8% of the overall population were aged 60+ in 2011 and this figure is expected to grow to 11.1% by 2025 [27]. About 60% of deaths in India are due to NCDs and the onset of NCDs is at least 10 years lower than developed countries [10]. This pattern of disability varies across states and by socioeconomic status [4, 28]. Over one-third of country’s population is living below the poverty line and health care quality and access is poor in poorer regions and among poorer people [29].

### Need for the study

In India, studies on disability are limited, largely due to the paucity of data. While the Census of India provides aggregate estimates of disability every 10 years, the National Sample Survey is the only data source that provides individual level data on disability, most recently in 2018. India is rapidly ageing and the disease patterns are changing rapidly. The onset of NCDs in India is at least 10 years lower than many developed countries [11]. Increasing disability is largely due to demographic and epidemiological transitions as well as socioeconomic and environmental causes. Though studies shows lower longevity among poorer individuals compared to richer individuals, little is known about the economic gradient of disability [30]. With its limited health care facilities and low coverage social security system, the poor and marginalised in India are likely to suffer most. In this context, the main objective of this paper is to estimate the economic gradient of the onset of seven types of disability in India.

### Data and methods

We obtained individual level data from the 76th round, 26.0 schedule of the National Sample Survey (NSS), conducted by the Ministry of Statistics and Programme Implementation, Govt. of India in 2018. The NSS is the official statistical system in the country that collects data on various socioeconomic and health issues through population-based surveys [17]. It used a nationally representative stratified two-stage sampling design. The NSS is the only data source in India that provides an opportunity to understand the onset of disability and its correlates. The 76th round covered 576,569 individuals from 118,152 households in India. A total of 106,894 disabled individuals were included in our analysis. The survey covered seven types of disability: locomotor, visual, hearing, speech, mental retardation, mental illness, and other disability. Additional file 1 presents the definition of each disability in the NSS survey. A person is considered to have a disability if he or she has restrictions or a lack of abilities to perform an activity in the manner or within the range considered normal for a human being. Persons with more than one disability type are considered to have multiple disabilities. The 76th round of the NSS was the first attempt to collect data on the prevalence of mental retardation and mental illness. The survey had a specific question regarding the age (in years) of onset of disability for those who reported to have any form of disability. Disability at age 0 was considered disability at birth.

## Methods

Descriptive statistics, disability rate, kernel density curves, Kaplan-Meier survival estimates, and multiple regression analyses were used in the analyses. Disability rate is defined as the number of disabled persons per 100,000 population. We examined the onset of disability at the 25th percentile, median, and 75th percentile. Kernel density is used to estimate the probability density function of age of onset of disability. The Kaplan-Meier (KM) survival function gives the probability that a person develops a particular disability after a given age (x). In other words, it is the probability that a person survives the age duration (up to age x) without the occurrence of that disability. The KM estimate of survival time *S(t)* is given by:

1 $$ S(t)={\prod}_{t_i\le t}^k\frac{\left({n}_i-{d}_i\right)}{n_i}, $$

where n_i_ is the number of individuals observed at time t_i_ and d_i_ is the number of individuals who experienced the disability at time t_i_. We tested for statistically significant differences in survival functions by monthly per capita consumption expenditure (MPCE) quintile using log rank tests.

The KM function was suitable because disability onset is a continuous time-to-event outcome and is relatively robust to competing events. A set of multiple linear regression equations are used to estimate the adjusted mean of the onset of disabilities. The regression equation in its general form is given as

2 $$ {Y}_i=\alpha +{\beta}_1{MCPE\ Quintile}_i+{\beta}_2{Sex}_i+{\beta}_3{Residence}_i+{\beta}_4{Education}_i+{\beta}_5{Religion}_i+{\beta}_6{Social\ Gorup}_i+{\beta}_7{Household\ Size}_i+{\varepsilon}_i $$

Where Y_i_ is the type of disability (outcome variable) for individual i and the β’s are the regression coefficients of independent variables monthly per capita consumption expenditure (MPCE) quintile (poorest, poorer, middle, richer and richest) which is an economic indicator measuring the economic well-being of the household and it is a continuous variable, sex (male/female), place of residence (rural/urban), education (below primary, middle or secondary, and secondary and above), religion (Hindu, Muslim, Others), social group (SC, ST, OBC, and others), and household size (1–28). ε_i_ is the error term in the regression model for individual i.

## Results

Table 1 presents the household characteristics of the overall surveyed population in India. The average household size was 4.3 persons and about 27% of the population was illiterate. The sex ratio (number of females per 1000 males) was 929 while the median age was 27 years. About 28% of the population were scheduled caste (SC)/scheduled tribe (ST) and 30.4% population were living in urban areas. The average MPCE was 2297 rupees (£25.20 in 2018). The average disability rate (per 100,000 population) in India was 2184 and it was highest for locomotor (1353), followed by hearing disability (296) and visual disability (234). The disability rate was lowest for mental illness (131) and all other disabilities (55).

**Table 1:** Sample Profile of surveyed population in India, 2018

Variables	Number/prevalence/percentage
Total Population Covered	576,569
Number of households	118,151
Average household size	4.3
Sex ratio (Number of females per 1000 males)	929
Percent Urban	30.4
Percent SC/ST	27.71
Median Age	27
**Education Attainment in percent**	
Illiterate	26.99
Up to Primary	29.11
Middle/Secody	26.23
Higher Secondary & above	17.67
Monthly per capita consumption expenditure (in Rupees)	2297
Number of disability cases	106,894
**Any disability rate (Per 100,000 population)**	2184
Locomotor disability rate	1353
Visual disability rate	234
Hearing disability rate	296
Speech disability rate	228
Mental retardation disability rate	158
Mental illness disability rate	131
Other disability rate	55

**Fig. 1:**
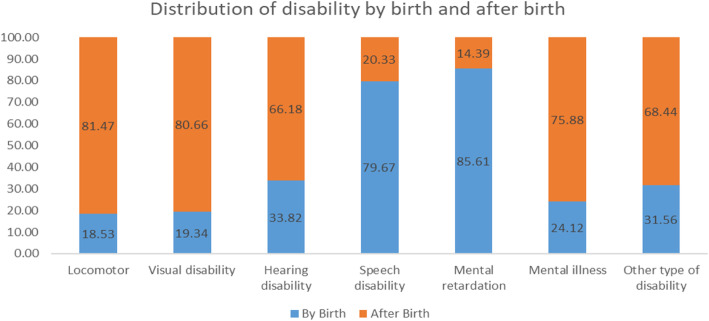
Distribution of disability by birth and after birth in India, 2018

Figure 1 shows the distribution of disabilities at birth and after birth in India. About 18.53% of locomotor disabilities and 19.34% of visual disabilities were present at birth. In contrast, 85.61% of individuals with mental retardation and 79.67% of individuals with a speech disability had their disability at birth.

Table 2, shows the distribution of onset of disability by MPCE quintile, sex, and residence for the seven types of disabilities in India. The estimates are presented at the 25th, 50th, and 75th percentiles of the distribution. The economic disparity of disability burden was strongest in the case of locomotor and speech disabilities. The 25th percentile of onset of locomotor disability was 8 years for the poorest MPCE quintile, 10 years for poorer and middle MPCE quintiles, 14 years for the rich MPCE quintile, and 24 years for richest MPCE quintile. Similarly, the median age of locomotor disability for people belonging to the poorest MPCE quintile in India was 39 years compared to 49 years for the richest MPCE quintile. In contrast, there are only small differences in the 75th percentile age at onset for locomotor disability across MPCE quintiles. Males had earlier age at onset of locomotor disability compared to females at each percentile of the distribution. The median age at onset of locomotor disability in rural areas is 4 years earlier than that of urban areas.

**Table 2:** Distribution of onset of disability (years) by MPCE quintile, sex and residence in India, 2018

	Locomotor			Visual			Hearing			Speech			Mental Retardation			Mental Illness			Other Disability^**a**^		
	P25	P50	P75	P25	P50	P75	P25	P50	P75	P25	P50	P75	P25	P50	P75	P25	P50	P75	P25	P50	P75
**MPCE Quintile**																					
Poorest	8	39	59	35	55	65	35	55	65	3	10	53	2	2	12	15	25	40	8	40	61
Poor	10	42	60	35	55	65	35	55	65	4	22	52	2	2	25	15	25	41	10	38	57
Middle	10	42	60	39	56	65	39	55	66	4	26.5	55	2	2	21	18	28	44	10	41	59
Rich	14	45	61	38	56	66	40	57	67	4	34	58	2	2	26	19	30	45	12	42	58
Richest	24	49	63	40	55	65	40	59	70	4	40	61	2	2	36	18	30	48	20	48	62
Total	12	44	60	38	55	65	38	56	67	4	29	56	2	2	25	17	28	45	13	44	60
***Sex***																					
Male	10	39	58	33	54	64	38	56	66	4	32	57	2	2	22	16	25	40	12	45	60
Female	19	50	63	41	57	65	40	56	68	3	21	56	2	2	32	19	31	48	14	43	59
Total	12	44	60	38	55	65	38	56	67	4	29	56	2	2	25	17	28	45	13	44	60
**Residence**																					
Rural	11	42	60	40	56	65	38	55	66	4	26	56	2	2	25	18	28	44	11	40.5	58
Urban	16	46	62	34	54	64	40	58	68	4	34	57	2	2	24	17	28	45	17	48	62
Total	12	44	60	38	55	65	38	56	67	4	29	56	2	2	25	17	28	45	13	44	60

^a^Other disability includes: Parkinson’s disease, multiple sclerosis, other chronic neurological conditions, hemophilia, thalassemia, sickle cell disease

Visual and hearing disability have similar patterns of onset. The median age at onset of visual and hearing disability across MPCE quintiles varies in a narrow range of 55 to 59 years. Onset of mental retardation disability occurs very early in life across MPCE quintiles, sex, and residence. For most disabilities, onset is earlier in males compared to females and in rural areas compared to urban areas. Notably, the distributions of the onset of hearing, speech, mental retardation, and mental illness disability are similar by residence.

Table 3 shows the adjusted mean onset of each disability from the linear regression. Controlling for socioeconomic factors, the age of onset of all disabilities in India increases with increasing MPCE quintile. For locomotor disabilities, for example, age of onset was 33.5 years (SE = 0.0000526) for people belonging to the poorest MPCE quintile compared to 40 (SE = 0.0000577) years for the richest MPCE quintile. The results show the strongest economic gradient for onset of speech and mental retardation disabilities, with adjusted mean differences between the poorest and the richest quintiles of 11 and 12 years respectively. Disability onset also showed gender differences, with generally earlier age at onset for males compared to females with the notable exception of speech disabilities. These differences were most pronounced for locomotor (5 year difference), mental illness (5 year difference), visual (4 year difference), and mental retardation (4 year difference) disabilities.

**Table 3:** Adjusted mean and standard error in age of onset (years) by type of disability in India, 2018

	Locomotor		Visual		Hearing		Speech		Mental Retardation		Mental Illness		Other Disability^**a**^	
	Mean	SE	Mean	SE	Mean	SE	Mean	SE	Mean	SE	Mean	SE	Mean	SE
**MPCE Quintile**														
Poorest	33.56	0.0000526	45.91	0.0000451	46.59	0.0000392	26.69	0.0000192	12.56	0.0000411	29.46	0.0000196	36.2	0.0000267
Poor	34.88	0.0000567	45.86	0.0000492	45.92	0.000042	29.12	0.0000211	17.73	0.0000437	29.51	0.0000207	34.87	0.000027
Middle	35.77	0.0000557	46.67	0.0000487	48.2	0.0000406	31.47	0.0000215	16.19	0.000044	32.11	0.0000204	36.38	0.0000257
Rich	36.96	0.0000576	46.52	0.0000509	49.45	0.0000412	34.04	0.0000234	17.94	0.0000473	32.87	0.0000208	37.04	0.0000271
Richest	39.74	0.0000577	46.23	0.0000512	50.86	0.0000398	37.5	0.0000247	24.41	0.0000486	34.45	0.0000207	41.71	0.0000289
**Gender**														
Male	33.78	0.0000351	44.44	0.00003	47.94	0.0000266	32.5	0.0000206	15.95	0.0000317	29.46	0.0000117	37.23	0.0000193
Female	38.75	0.0000348	48.17	0.0000301	48.49	0.0000267	30.9	0.0000207	19.62	0.0000317	34.06	0.0000121	37.26	0.0000201
**Residence**														
Rural	35.28	0.0000304	46.98	0.0000258	47.46	0.0000225	30.79	0.0000161	16.27	0.000025	31.35	0.0000128	36.19	0.0000156
Urban	38.19	0.000047	44.53	0.0000402	49.89	0.0000329	33.9	0.0000285	21.03	0.000045	32.41	0.0000192	39.66	0.0000243
**Education**														
Below Primary	42.09	0.0000212	52.35	0.0000111	52.36	0.0000161	28.88	0.0000131	13.08	0.0000205	32.86	0.0000131	36.38	0.0000176
Middle/Secondary	29.23	0.0000334	39.54	0.000016	41.11	0.0000242	32.91	0.0000193	20.29	0.0000296	29.65	0.0000197	37.27	0.0000263
Secondary & above	27.64	0.0000448	36.77	0.0000185	45.57	0.0000302	39.04	0.0000234	28.64	0.0000365	30.92	0.0000244	39.95	0.0000331
**Religion**														
Hindu	36.44	0.0000286	46.68	0.0000245	48.69	0.0000207	31.95	0.0000165	18.57	0.0000253	31.88	0.0000118	37.57	0.0000111
Muslim	33.36	0.0000644	44.35	0.0000543	44.9	0.0000464	29.67	0.0000327	12.85	0.0000526	30.03	0.0000262	31.42	0.000022
Others	39.79	0.0001173	44.47	0.0000991	49.89	0.0000866	34.11	0.0000713	17.97	0.0001003	33.09	0.0000494	48.65	0.0000463
**Social group**														
ST	35.99	0.0000746	46.55	0.0000666	45.41	0.0000562	27.65	0.0000414	22.75	0.0000614	30.04	0.0000318	42.37	0.0000412
SC	33.71	0.000056	45.13	0.0000491	45.86	0.0000405	31.92	0.0000305	17.37	0.0000458	30.55	0.0000227	35.38	0.0000241
OBC	35.08	0.0000376	46.86	0.000033	48.51	0.0000276	31.2	0.0000203	14.89	0.0000312	31.33	0.0000152	35.8	0.0000166
Others	39.75	0.0000494	45.84	0.000043	50.25	0.0000358	33.9	0.0000294	21	0.0000459	33.59	0.0000199	39.2	0.0000287
**Household Size**														
1–4	36.6	0.000041	45.17	0.000035	48.39	0.0000298	32.96	0.0000241	19.84	0.0000374	32.11	0.000017	37.66	0.0000224
5–8	36.11	0.0000362	46.7	0.0000304	47.97	0.0000267	31.37	0.0000196	15.84	0.0000304	31.47	0.0000152	37.61	0.0000186
9–28	34.78	0.0000821	48.06	0.0000687	48.64	0.0000601	28.77	0.000041	18.93	0.0000678	30.99	0.0000333	33.77	0.0000401

^a^Other disability includes: Parkinson’s disease, multiple sclerosis, other chronic neurological conditions, hemophilia, thalassemia, sickle cell disease

Adjusted for other socioeconomic indicators, we observed gaps in the onset of disability between social groups. These differences were most pronounced for mental retardation with mean onset at age 22.75 for ST (SE = 0.0000614), 17.37 for SC (SE = 0.0000458), and 14.89 for OBC (SE = 0.0000312). However, this pattern was reversed for hearing disability with mean onset at age 45.41 for ST (SE = 0.0000562), 45.86 for SC (SE = 0.0000405), and 48.51 for OBC (SE = 0.0000276). Across all disabilities, Muslims showed lower mean age of disability onset compared to Hindus. The most pronounced difference was mental retardation with mean age at onset of 12.85 years (SE = 0.0000253) for Muslims compared to 18.57 years (SE = 0.0000253) for Hindus. Education did not show a clear gradient across disability types. For locomotor and visual disabilities, lower education had later onset than higher education, while for speech and mental retardation it was reversed. Other disabilities such as hearing and mental illness had the lowest age of onset for middle/secondary education, while primary education and secondary and above had higher ages of onset. There were no major differences in age of onset across disabilities by household size.

**Fig. 2:**
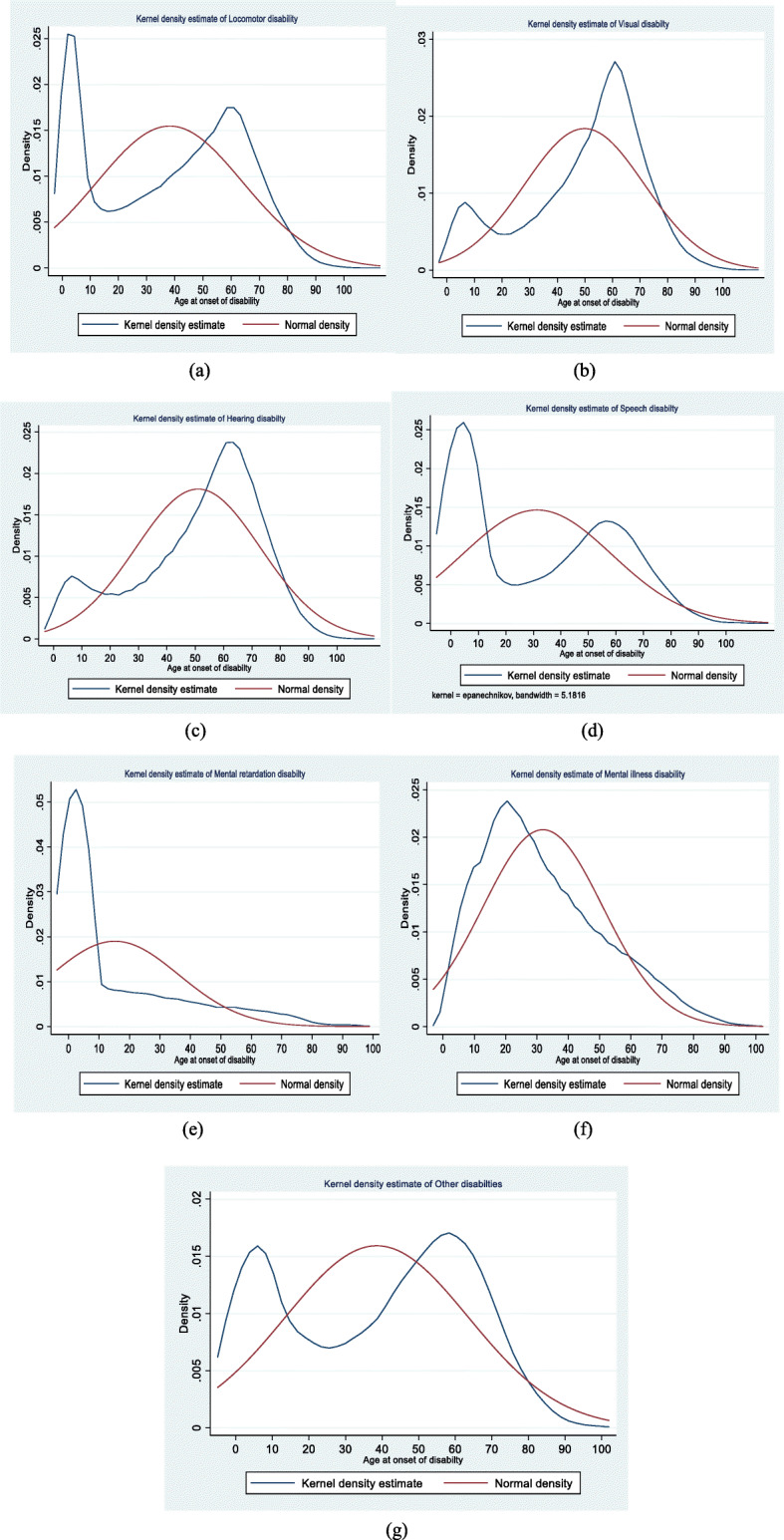
Kernel density estimates of (**a**) locomotor (**b**) visual (**c**) hearing (**d**) speech (**e**) mental retardation (**f**) mental illness and (**g**) other disabilities in India, 2018

Figure 2 shows the kernel density estimates of onset of disability across age groups for the seven types of disabilities. The purpose of the kernel density is to reflect the proportion of disability in each age group. Locomotor disability onset has two peaks, at ages 0 and 60, with a minimum at age 25 (Fig. 2a). The normal curve shows an expected peak of onset of disability by the age 36 after which it starts declining. The kernel density plots of visual (Fig. 2b) and hearing (Fig. 2c) disability show that onset is likely to occur in the early to mid-60s, each with a smaller peak in early childhood about age 6 consisting of about 1% of cases. Speech disability also has a bimodal kernel density plot with a peak before 5 years old and a smaller peak in the mid-50s. The mental retardation and mental illness plots are both unimodal with peaks in early childhood and early adulthood respectively. The low age peak for mental retardation is expected given the high proportion of cases present at birth. For all other disabilities similarly sized peaks occurred in early childhood and about age 60.

**Fig. 3:**
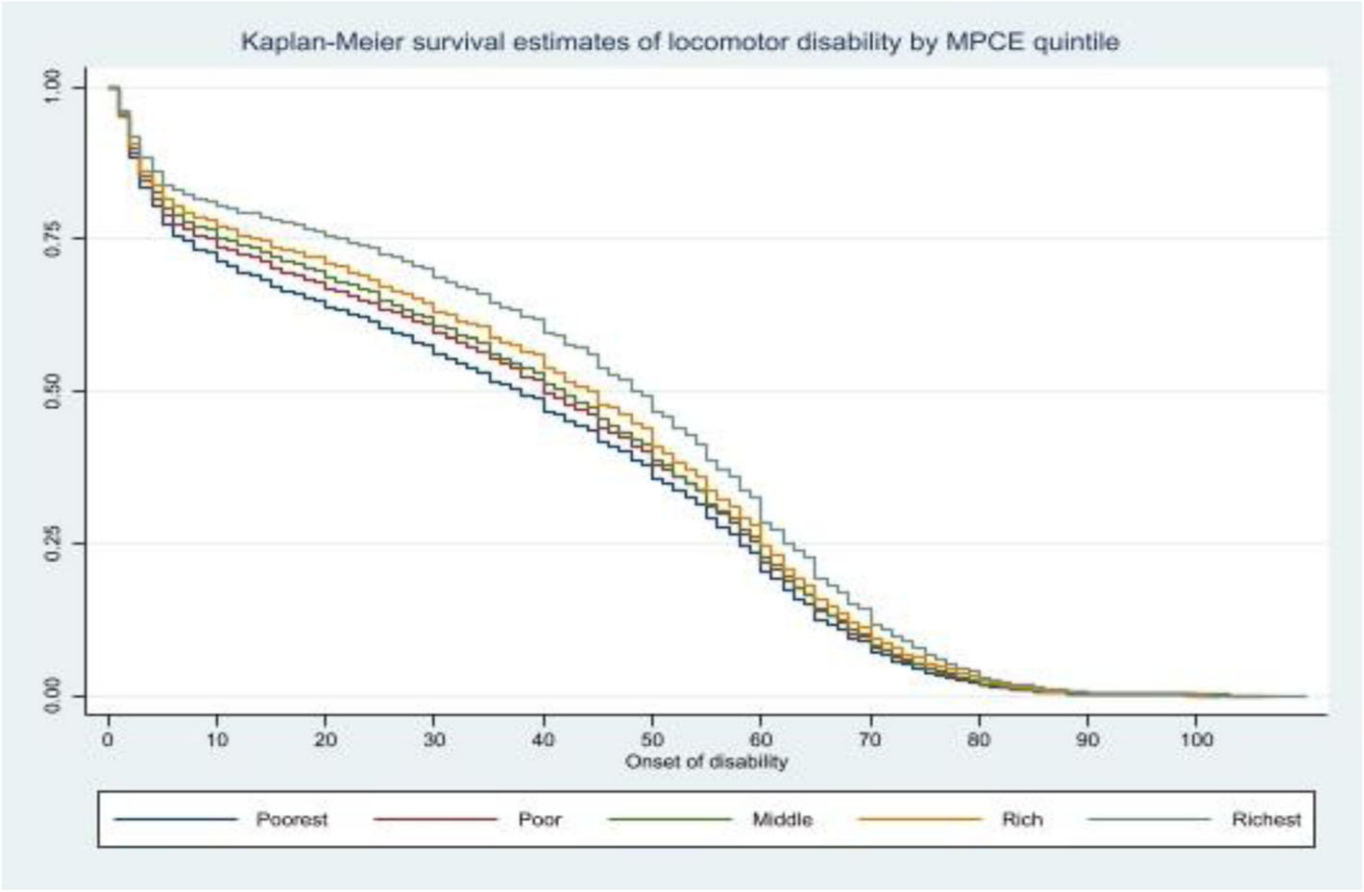
Kaplan-Meier survival estimate of locomotor by MPCE quintile in India, 2018

Figure 3 shows the KM survival curves for all types of disabilities by MPCE quintile in India. The curves differed by MPCE quintile. Log rank tests showed people in the poorest MPCE quintile had a higher probability of occurrence of disabilities at earlier ages compared to the richest MPCE quintile. With increasing MPCE quintile, the age of onset of disability increases in a type of dose-response relationship. Locomotor disability had the highest probability of onset among working age groups in India.

**Fig. 4:**
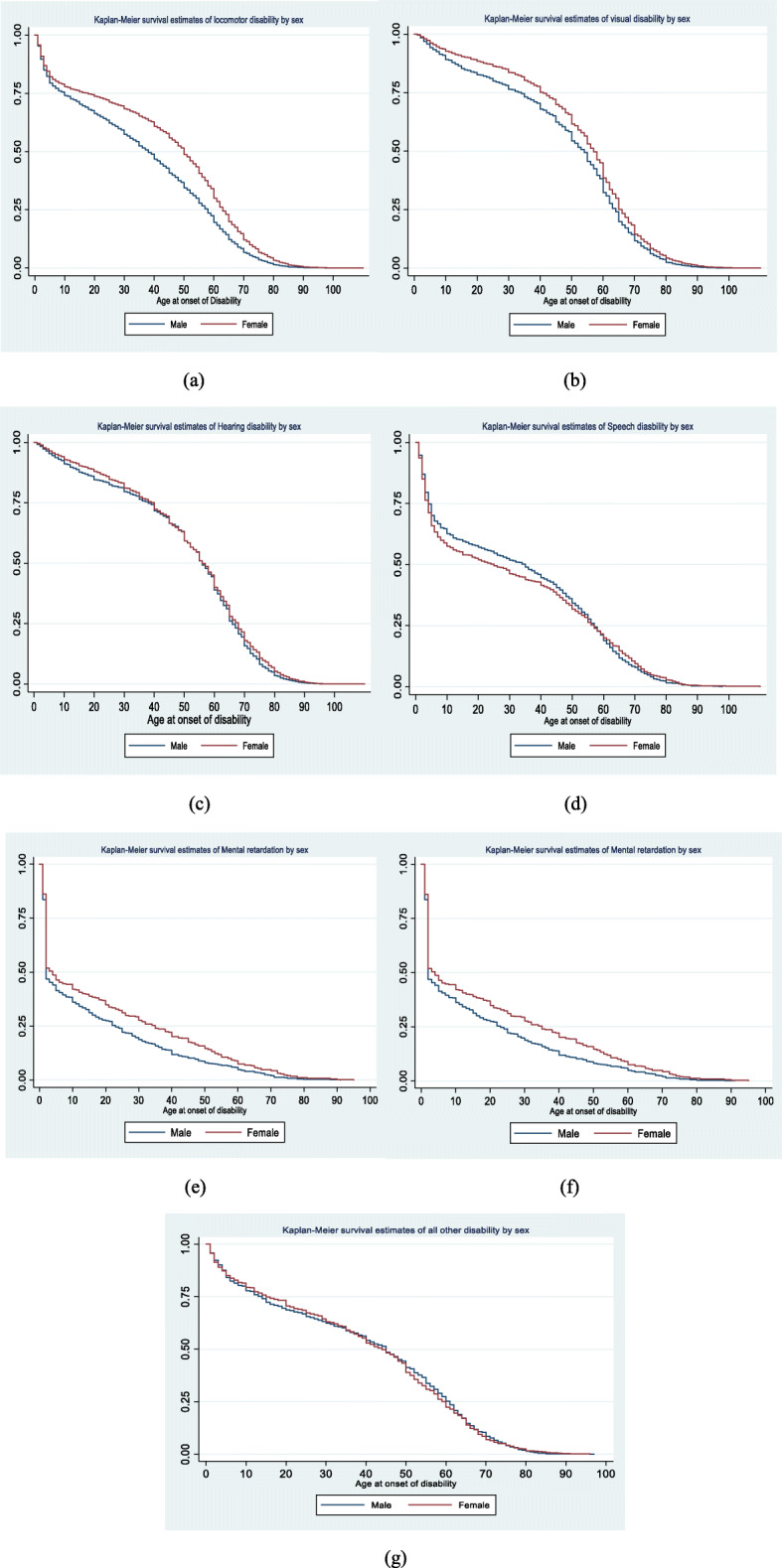
Kaplan-Meier survival estimate of (**a**) locomotor (**b**) visual (**c**) hearing (**d**) speech (**e**) mental retardation (**f**) mental illness and (**g**) other disabilities by sex in India, 2018

Figure 4 presents the KM survival curves by type of disability stratified by sex. The KM survival curves for males remained below those for females across all ages for locomotor, visual, mental retardation, and mental illness disability, reflecting earlier onset of these disabilities in males. There were no substantial differences in the survival curves for hearing, speech, and other disabilities by sex.

## Discussion

To our knowledge, this is the first study to estimate the onset of seven major disabilities by socioeconomic and demographic characteristics in India. It improves our understanding of how the age of onset of disability is distributed over the life course among different types of disabilities as well as the association between economic and socioeconomic variation in age of onset of disabilities.

We show that the age of onset of disability varies by type of disability in India. Among the seven types of disabilities, the median age of onset of disability was lowest for mental retardation (2 years) and highest for hearing (56 years). Mental retardation and mental illness appear at earlier ages compared to other disabilities, some of which primarily appear in later life including hearing and visual disabilities. Several disabilities also show bimodal patterns of age of onset with the highest proportion of burden occurring in early childhood and old age. We observed a clear economic gradient of onset of disability both overall and by specific type of disability. This gradient was strongest for locomotor and speech disabilities. Controlling for sociodemographic correlates, people in poorer strata are more prone to experience early age of onset of locomotor and speech disability, at least 7 years before the richest. Differences by sex were also seen with age of onset of locomotor and visual disability occurring earlier among males than females while speech and other disabilities had earlier onset among females. Notably, rural populations were more likely to have lower age of onset of disability compared to urban populations.

When we look into the interrelationship between disability and economic characteristics, enduring disability throughout the life course may lead to poverty and social exclusion, in part brought about by the burden of familial financial dependency. Disabled individuals are more vulnerable to economic disempowerment than non-disabled individuals [31]. Our findings are consistent with the extant literature. Prior studies suggest higher disability among the poor [2, 32]. Possible explanations include lack of adequate health care quality and access. The higher proportion of disability among males compared to females is also consistent with the literature [33]. This disparity may be due to higher NCD burden among males compared to females. Males are more likely to be involved in accidents and incur injuries compared to females [34]. This study’s findings regarding age of onset of disability differences by socioeconomic and demographic factors corroborates previous literature. Previous study results show that the age of onset of disability is associated with geographical region, education level, and wealth quintile [14, 35]. Children and older adults are more prone to diseases and health complications and so age of onset of disability was higher at younger and older ages [21]. The high locomotor disability rate in rural populations is coupled with earlier age of onset of disability [2, 32, 36].

This study has several limitations. First, the age of onset of disability is self-reported and not clinically validated. Less than one-third of disabled persons had a medical certificate while the majority of disabled persons lacked such documentation. Second, we did not explore the causes of disability such as diseases, accidents, injuries, genetics etc. Third, the state level variation across types of disabilities could not be analysed due to insufficient sample size.

## Conclusion

Despite these limitations, this study provides numerical estimates on age of onset of disability that would be helpful for national and state planning. Though national and state governments provide some benefits to disabled persons (reserved spots in education and job placements as well as pension benefits for disabled persons), there is limited health provisioning for the disabled population in India. Providing free health care facilities, universal health insurance coverage for disabled, and economic security for disabled persons could mitigate suffering. Different disabilities require different accommodations and this study illuminates how burden is shared unequally across populations and through the life course, which may help guide policymakers and public health practitioners focus their efforts more effectively.

## Supplementary Information

**Additional file 1.**

## Data Availability

Available in the public domain http://mospi.nic.in/.

## Abbreviations

NCD: Non communicable disease
NSS: National sample survey
MPCE: Monthly per capita consumption expenditure
SC: Schedule caste
ST: Schedule tribe
OBC: Other backward classes
SE: Standard error
KM: Kaplan Meier
WHO: World health organization

## References

[1] WHO (2018). Disabilities.

[2] Kisioglu AN, Uskun E, Ozturk M (2003). Socio-demographical examinations on disability prevalence and rehabilitation status in southwest of Turkey. Disabil Rehabil.

[3] Mitra S, Posarac A, Vick B (2013). Disability and poverty in developing countries: a multidimensional study. World Dev.

[4] Dandona R, Pandey A, George S, Kumar GA, Dandona L (2019). India’s disability estimates: limitations and way forward. PLoS One.

[5] The United Nations (2006). Convention on the rights of persons with disabilities. Treaty Series.

[6] Márton SM, Polk G, Fiala DRC (2013). Convention on the rights of persons with disabilities.

[7] United Nations. Goal 3: ensure healthy lives and promote well-being for all at all ages, vol. 2030: U. Nations, Transforming our World: The Agenda; 2015. p. 22. https://www.un.org/sustainabledevelopment/health/.

[8] United Nations. The Sustainable Development Goals [cited 2019 22 July]. https://www.un.org/sustainabledevelopment/sustainable-development-goals/.

[9] Unwin N, Alberti KGMM (2006). Chronic non-communicable diseases. Ann Trop Med Parasitol.

[10] Nethan S, Sinha D, Mehrotra R (2017). Non communicable disease risk factors and their trends in India. Asian Pac J Cancer Prev.

[11] Arokiasamy P (2018). India's escalating burden of non-communicable diseases. Lancet Glob Health.

[12] Melzer D, McWilliams B, Brayne C, Johnson T, Bond J (2000). Socioeconomic status and the expectation of disability in old age: estimates for England. J Epidemiol Community Health.

[13] Mitra S, Sambamoorthi U. Employment of persons with disabilities: evidence from the National Sample Survey. Econ Polit Wkly. 2006;21:199–203.

[14] Elwan A (1999). Poverty and disability: a survey of the literature.

[15] Burchardt, T. (2003). Being and becoming: social exclusion and the onset of disability. CASE report. London: London School of Economics/ESRC Research Centre for Analysis of Social Exclusion (CASE).

[16] Minkler M, Fuller-Thomson E, Guralnik JM (2006). Gradient of disability across the socioeconomic spectrum in the United States. N Engl J Med.

[17] Ministry of Statistics and Planning Implementation (2018). Report on Person with disabilities in India.

[18] Power C, Li L (2000). Cohort study of birth weight, mortality, and disability. BMJ.

[19] De Vries BB, Pfundt R, Leisink M, Koolen DA, Vissers LE, Janssen IM (2005). Diagnostic genome profiling in mental retardation. Am J Hum Genet.

[20] Curry CJ, Stevenson RE, Aughton D, Byrne J, Carey JC, Cassidy S (1997). Evaluation of mental retardation: recommendations of a consensus conference. Am J Med Genet.

[21] Brito KQD, de Menezes TN, de Olinda RA. Functional disability and socioeconomic and demographic factors in elderly. Revista Brasileira Enfermagem. 2015;68(4):633-40. 10.1590/0034-7167.2015680409i 26422034

[22] Bjelland MJ, Bruyere SM, Von Schrader S, Houtenville AJ, Ruiz-Quintanilla A, Webber DA (2010). Age and disability employment discrimination: occupational rehabilitation implications. J Occup Rehabil.

[23] Adamson J, Hunt K, Ebrahim S (2003). Socioeconomic position, occupational exposures, and gender: the relation with locomotor disability in early old age. J Epidemiol Community Health.

[24] WHO and World Bank (2011). World Report on Disability.

[25] Saikia N, Bora JK, Jasilionis D, Shkolnikov VM (2016). Disability divides in India: evidence from the 2011 census. PLoS One.

[26] Census of India. Data on Disability, vol. 2011. 2017, New Delhi: Office of the Registrar General & Census Commissioner. Available at http://www.disabilityaffairs.gov.in/upload/upload/files/files/disabilityinindia2011data.pdf.

[27] United Nations Department of Economic and Social Affairs, Population Division (2008). World Population Prospects (2008 Revision).

[28] Awasthi A, Pandey CM, Dubey M, Rastogi S (2017). Trends, prospects and deprivation index of disability in India: evidences from census 2001 and 2011. Disability Health J.

[29] Dash A, Mohanty SK (2019). Do poor people in the poorer states pay more for healthcare in India?. BMC Public Health.

[30] Kumari M, Mohanty SK (2020). Caste, religion and regional differentials in life expectancy at birth in India: cross-sectional estimates from recent National Family Health Survey. BMJ Open.

[31] Elwan A (1999). Poverty and disability: a survey of the literature.

[32] Beydoun MA, Popkin BM (2005). The impact of socio-economic factors on functional status decline among community-dwelling older adults in China. Soc Sci Med.

[33] Mishra RS, Mishra R, Mohanty SK. Gender differential and regional disparity of disability-free life-expectancy among disable in India. Clin Epidemiol Glob Health. 2020;8(3):818-27.

[34] Sorenson SB (2011). Gender disparities in injury mortality: consistent, persistent, and larger than you'd think. Am J Public Health.

[35] Mandemakers JJ, Monden CW (2010). Does education buffer the impact of disability on psychological distress?. Soc Sci Med.

[36] Choi H, Marks NF (2008). Marital conflict, depressive symptoms, and functional impairment. J Marriage Fam.

