# Supplementary material for: Economic gradient of onset of disability in India
Source: BMC Public Health. 2021 Apr 21;21:769. doi: 10.1186/s12889-021-10826-5 (PMC8061006; doi:10.1186/s12889-021-10826-5)
Supplement: Supplementary file 1 — Additional file 1. [file 12889_2021_10826_MOESM1_ESM.docx]

**Additional file 1**

The definition on disability used in the NSS is given below.

1. **Locomotor disability:** Those having difficulty in using hands, fingers, toes, in body movement or whether had loss of sensation in the body due to paralysis, leprosy, other reasons or whether having deformity of the body part (s) like hunch back, dwarfism, deformity due to leprosy, caused by acid attack, etc.
2. **Visual disability:** Whether having difficulty in seeing, counting fingers of hand from a distance of 10 feet (with spectacles, if using, and both eyes taken together)
3. **Hearing disability:** Whether having difficulty in hearing day to day conversational speech (without hearing aid, if using, and both ears taken together)
4. **Speech disability:** Whether having difficulty in speech (unable to speak like normal person/ speech not comprehensible, including laryngectomy, aphasia)
5. **Mental retardation disability:** Whether having difficulty in understanding/ comprehension or communicating (in doing daily activities or required in reasoning, making decision, remembering, learning, problem solving)
6. **Mental illness disability:** Whether having unnecessary and excessive worry and anxiety, repetitive behaviour/ thoughts, changes of mood or mood swings, talking/ laughing to self, staring in space Or Whether having unusual experiences of hearing voices, seeing visions, strange smell or sensation or strange taste Or Whether having unusual behaviour or difficulty in social interactions and adaptability
7. **Other disability:** Whether having any of the following: Parkinson's disease, multiple sclerosis, other chronic neurological conditions, haemophilia, thalassemia, sickle cell disease
